# Interpretable Learning Approaches in Resting-State Functional Connectivity Analysis: The Case of Autism Spectrum Disorder

**DOI:** 10.1155/2020/1394830

**Published:** 2020-05-18

**Authors:** Jinlong Hu, Lijie Cao, Tenghui Li, Bin Liao, Shoubin Dong, Ping Li

**Affiliations:** ^1^School of Computer Science and Engineering, South China University of Technology, Guangzhou, China; ^2^Communication and Computer Network Laboratory of Guangdong, South China University of Technology, Guangzhou, China; ^3^College of Mathematics and Informatics, South China Agricultural University, Guangzhou, China; ^4^Faculty of Humanities, The Hong Kong Polytechnic University, Hong Kong, China

## Abstract

Deep neural networks have recently been applied to the study of brain disorders such as autism spectrum disorder (ASD) with great success. However, the internal logics of these networks are difficult to interpret, especially with regard to how specific network architecture decisions are made. In this paper, we study an interpretable neural network model as a method to identify ASD participants from functional magnetic resonance imaging (fMRI) data and interpret results of the model in a precise and consistent manner. First, we propose an interpretable fully connected neural network (FCNN) to classify two groups, ASD versus healthy controls (HC), based on input data from resting-state functional connectivity (rsFC) between regions of interests (ROIs). The proposed FCNN model is a piecewise linear neural network (PLNN) which uses piecewise linear function LeakyReLU as its activation function. We experimentally compared the FCNN model against widely used classification models including support vector machine (SVM), random forest, and two new classes of deep neural network models in a large dataset containing 871 subjects from ABIDE I database. The results show the proposed FCNN model achieves the highest classification accuracy. Second, we further propose an interpreting method which could explain the trained model precisely with a precise linear formula for each input sample and decision features which contributed most to the classification of ASD versus HC participants in the model. We also discuss the implications of our proposed approach for fMRI data classification and interpretation.

## 1. Introduction

Autism spectrum disorder (ASD) is a subtype of extensive developmental disorder which is characterized by reciprocal social communication impairment as well as repetitive, restricted, and stereotyped behaviors [1]. The cause of ASD is uncertain, and the diagnosis is often difficult since the expressions of ASD symptoms are diverse and may vary over the course of development [2]. Functional magnetic resonance imaging (fMRI) is one of the most widespread approaches which is noninvasive and useful for understanding brain function [3]. fMRI has been applied recently for distinguishing ASD patients from healthy controls, and various machine learning methods have been used to analyze fMRI data of brain disorder [4–7]. However, so far, it has been challenging to analyze fMRI data for brain disorder due to the data characteristics such as high dimensionality, structural complexity, nonlinear separability, and the sequential changes of traceable signals in each voxel [8].

Given the excellent learning capability and classification performance in many domains, deep learning methods have been recently applied to fMRI data from ASD patients [9–14]. Sólon et al. [9] investigated the patterns of functional connectivity that help to identify ASD participants from functional brain imaging data. They used stacked denoising autoencoders for the unsupervised pretraining stage to extract a low-dimensional version from the ABIDE database and then applied the encoder weights to a multilayer perceptron for classification. The ABIDE (Autism Brain Imaging Data Exchange) database [15] contains a rich set of fMRI data that has aggregated functional and structural brain imaging data collected from multisite around the world (see Section 2.1 below for details). Guo et al. [10] stacked multiple sparse autoencoders for data dimension reduction and developed a feature selection method to select features with high discriminative power. Then, they used a softmax regression on top of the stacked sparse autoencoders for data classification. Eslami et al. [11] used an autoencoder and a single-layer perceptron to extract lower dimensional features, and the trained perceptron is used for the final round of classification. Brown et al. [12] proposed an element-wise layer based on BrainNetCNN [16] and used anatomically informed, data dependent, prior to regularize the weights of the layer.

Researchers are also trying to explain these models, by analyzing the discriminative features or potential neuroimaging biomarkers that contribute to the classification of ASD from healthy controls. Li et al. [17] trained a deep neural network to classify 3D fMRI volumes, developed a frequency-normalized sampling method to replace a ROI of the original image with the sampling data, and put it in the trained model to get a new prediction. Based on the different predicting performance, they used a statistical method to interpret the importance of the ROI. In the study of discovering imaging biomarkers for ASD [18], they went beyond looking at only individual features by using Shapley value explanation on interactive features' prediction power analysis. Guo et al. [10] proposed a deep neural network with a feature selection method from multiple trained sparse autoencoders, then developed Fisher's score-based biomarker identification method for their deep neural network using the rs-fMRI dataset in ABIDE I. These approaches all led to useful insights into the mechanism of deep learning models. However, such deep and nonlinear models are usually constructed as black boxes with complex network structure and hidden internal logic and are difficult to interpret with regard to how architecture decisions are consistently made by researchers [19].

In this study, we introduce an interpretable learning approach for resting-state functional connectivity analysis. We firstly propose an interpretable neural network model to distinguish between ASD participants and healthy controls (HC) based on resting-state functional connectivity (rsFC) of each subject. The proposed model is an interpretable fully connected neural network (FCNN), which uses piecewise linear function LeakyReLU as its activation function. It is a fully connected neural network including two hidden layers, input layer and output layer. Further, the proposed model is a piecewise linear neural network (PLNN) [20], which is mathematically equivalent to a set of local linear classifiers and could be interpreted precisely and consistently [19]. Secondly, taking advantage of the interpretation of PLNN, we propose an interpretable method which could explain the trained classification model with a precise linear formula for each input sample and the decision features which contribute most to classify ASD versus HC in the model.

We experimentally compared the proposed FCNN model against widely used benchmark models including SVM, random forest (RF), and two new neural network models in classifying data from the multisite ABIDE I database [15]. The proposed FCNN model, based on input data from rsFC between regions of interests (ROIs) accord to the AAL atlas [21], achieved the highest accuracy 69.81% in the large dataset containing 871 subjects (403 ASD patients and 468 healthy controls). We also explained the most important features in the model.

## 2. Dataset and Preprocessing

### 2.1. Dataset

We chose the dataset from the Autism Brain Imaging Data Exchange (ABIDE) initiative [15] to confirm the approach proposed in this study. The ABIDE initiative has aggregated functional and structural brain imaging data collected from multiple sites around the world. The dataset used in this study contained 871 subjects acquired from 17 acquisition sites with different imaging protocols that met the imaging quality and phenotypic information criteria [22]. This dataset includes 403 individuals suffering from ASD and 468 healthy controls (HC).

### 2.2. Preprocessing

We downloaded the preprocessed resting-state fMRI data from the Preprocessed Connectomes Project (PCP) (http://preprocessed-connectomes-project.org/abide/download.html). The data [23] was preprocessed by the Configurable Pipeline for the Analysis of Connectomes (CPAC) pipeline that included the following procedure: slice timing correction, motion realignment, intensity normalization, regression of nuisance signals, band-pass filtering (0.01-0.1 Hz), and registration of fMRI images to standard anatomical space (MNI152). The detailed description of pipeline can be found at http://preprocessed-connectomes-project.org/abide/Pipelines.html. The data was parcellated into 116 regions of interests (ROIs) using the AAL atlas [21].

## 3. Proposed Approach

**Figure 1:**
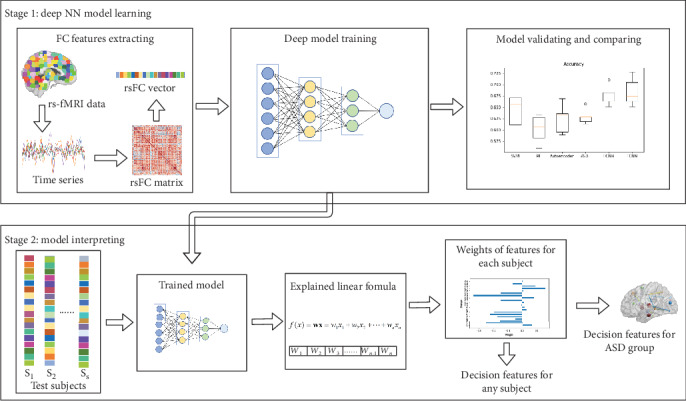
Flow chart of the proposed approach: learning and interpreting model on resting-state fMRI data.

The flow chart of the proposed interpretable learning approach is shown in Figure 1. First, we propose the FCNN model for classifying ASD and healthy participants, including extracting the rsFC features, training the FCNN model, and validating the model. Second, we interpret the trained model with an easily explained linear formula for each subject, identifying the decision rsFC features for the ASD group from the data.

### 3.1. Feature Extraction

The resting-state fMRI data was preprocessed as described in Section 2. The brain was parcellated into 116 regions of interests (ROIs) according to the AAL atlas [21]. Then, the mean time series of each ROI was extracted for each subject, and the rsFCs between ROIs were measured by computing Pearson's correlation coefficient of the extracted time series. A 116 × 116 connectivity matrix was constructed for each subject, respectively.

Fisher transformation was applied to the connectivity matrices to improve normality. The upper triangle values were then extracted and flattened into vectors, with the dimension of the feature vector which is (116 × (116 − 1))/2 = 6670.

### 3.2. FCNN Model

**Figure 2:**
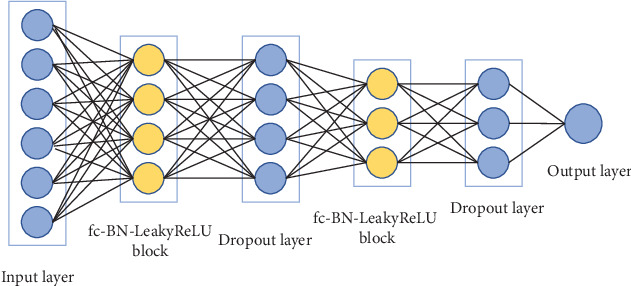
The architecture of the proposed FCNN model.

The architecture of the proposed FCNN model is shown in Figure 2. The FCNN is a fully connected neural network and a piecewise linear neural network (PLNN), where the PLNN is a deep neural network in which the nonlinear activation function is a piecewise linear function with a constant number of pieces [20].

The FCNN model contains two fc-BN-LeakyReLU blocks, where the fc-BN-LeakyReLU block consists of a fully connected (fc) layer followed by a Batch Normalization (BN) layer and LeakyReLU activation function.

LeakyReLU is a variant of rectified linear unit (ReLU) [24] which allows a small, positive gradient when the unit is not active [25]. For each hidden neuron *u*, LeakyReLU is defined as

(1) $$\begin{array}{c} f\left(u\right)=\left\{\begin{array}{c} u,\;u\geq 0,\\ \alpha u,\;u<0, \end{array}\right. \end{array}$$

where *α* represents slope coefficient. LeakyReLU is clearly a piecewise linear function.

In this study, for simplicity and clarity, we regarded a fc-BN-LeakyReLU block as a hidden layer. For a model with *L* layers, a fc layer can be formulated as

(2) $$\begin{array}{c} z^{\left(l+1\right)}=W^{\left(l\right)}a^{\left(l\right)}+b^{\left(l\right)}, \end{array}$$

where *l* ∈ {1, ⋯, *L* − 1}; suppose there are *n* neurons in layer *l* and *m* neurons in layer *l* + 1, *W*^(*l*)^ is *m* × *n* weight matrix, *b*^(*l*)^ is *m* × 1 bias vector, and *a*^(*l*)^ will be in Equation (3).

Then, the fc-BN-LeakyReLU block can be written as

(3) $$\begin{array}{c} a^{\left(l\right)}=f\left(\text{BN}\left(z^{\left(l\right)}\right)\right), \end{array}$$

where *l* ∈ {2, ⋯, *L* − 1} are hidden layers, *f*(∙) is the LeakyReLU function, explicitly, and *a*^(1)^ is the input instance *x*.

The sigmoid function is applied on the output layer to predict the probability of any given participant being an ASD patient. The number of units (nodes) is 6670, 64, 32, and 1, respectively, for input layer, two fully connected layers, and output layer. The dropout layer is added to avoid data overfitting, and the loss function uses binary cross entropy.

### 3.3. Interpreting Method

We interpret the trained neural network model with two stages: (i) computing the decision boundary of a fixed instance and the weight of features in linear formula for the instance and (ii) extracting and analyzing decision features of the trained model in the ASD group level.

In the first stage, we computed the decision boundary of a fixed instance *x*.

For each hidden neuron *u*, BN can be formulated as

(4) $$\begin{array}{c} y=\frac{\gamma }{\sqrt{\text{Var}\left[u\right]+\epsilon }}∙u+\left(\beta -\frac{\gamma \text{E}\left[u\right]}{\sqrt{\text{Var}\left[u\right]+\epsilon }}\right), \end{array}$$

where *γ* and *β* are learned parameters [26]. In the test phase of the model, Var[*u*] and *E*[*u*] are fixed, so Equation (4) can be regarded as a linear function.

As shown in Equation (1), for hidden neurons with LeakyReLU activation function, there are two kinds of activation status that each corresponds to a corresponding linear function where the mapping relationship between *f*(*u*) and *u* is linear. And it is proved that for a fixed PLNN model and a fixed instance *x*, the output of model *F*(*x*) on an input *x* can be seen as a linear classifier [19], which can be formulated as

(5) $$\begin{array}{c} F\left(x\right)=\text{sigmoid}\left(\hat{W}x+\hat{b}\right), \end{array}$$

where $\hat{W}=\prod_{h=0}^{L-3}{\tilde{W}}^{\left(L-1-h\right)}W^{\left(1\right)}$ is the coefficient vector of *x* and $\hat{b}$ is the constant intercept. For a fixed input instance *x*, *F*(*x*) is a linear classifier whose decision boundary is explicitly defined by $\hat{W}x+\hat{b}$. Therefore, $\hat{W}$ are weights assigned to the features of *x*.

As for FCNN, we computed $\tilde{W}$ as follows: since BN can be regarded as a linear function in the test phase of model as discussed above, the Equation (3) can be rewritten as

(6) $$\begin{array}{c} a^{\left(l\right)}=f\left({\tilde{\gamma }}^{\left(l\right)}\circ z^{\left(l\right)}+{\tilde{\beta }}^{\left(l\right)}\right), \end{array}$$

where ${\tilde{\gamma }}^{\left(l\right)}$ is the constant slope, ${\tilde{\beta }}^{\left(l\right)}$ is the constant intercept, for all *l* ∈ {2, ⋯, *L* − 1}. Since *f*(∙) is the piecewise linear activation function, Equation (6) can be rewritten as

(7) $$\begin{array}{c} a^{\left(l\right)}=r^{\left(l\right)}\circ \left({\tilde{\gamma }}^{\left(l\right)}\circ z^{\left(l\right)}+{\tilde{\beta }}^{\left(l\right)}\right)+t^{\left(l\right)}, \end{array}$$

where *r*^(*l*)^ is the constant slope and *t*^(*l*)^ is the constant intercept. By plugging Equation (7) into Equation (2), we rewrite *z*^(*l* + 1)^ as

(8) $$\begin{array}{c} z^{\left(l+1\right)}=W^{\left(l\right)}\left(r^{\left(l\right)}\circ \left({\tilde{\gamma }}^{\left(l\right)}\circ z^{\left(l\right)}+{\tilde{\beta }}^{\left(l\right)}\right)+t^{\left(l\right)}\right)+b^{\left(l\right)}={\tilde{W}}^{\left(l\right)}z^{\left(l\right)}+{\tilde{b}}^{\left(l\right)}, \end{array}$$

where ${\tilde{W}}^{\left(l\right)}=W^{\left(l\right)}\circ r^{\left(l\right)}\circ {\tilde{\gamma }}^{\left(l\right)}$ is an extended version of the Hadamard product.

In the second stage, based on the weights $\hat{W}$ for features of each test instance *x*, we could get the top *K* features with the highest weight. Then, we count the number of occurrences *n*^*f*^ of feature *f* in the top-k-feature-set from all the instances. By setting a threshold on *n*^*f*^, we can get decision feature set *F* which contributes most to classify ASD versus HC in the model.

The whole flow of the interpreting method is formulated as in Algorithm 1. We firstly obtain the top-k-feature-set *F*_*K*_^*X*^ for each instance *x*, and then, we obtain the decision feature set *F* by selecting the feature *f* whose occurrence number as a percentage of total instances is greater to the parameter *ε*. Meanwhile, we could also get the weights of all features for any specified test instance, which could help to explain the decision made by the trained model for the instance.

## 4. Classification Experiments

With the above approach and the model architecture, we conducted experiments on the ABIDE I dataset with 871 subjects and applied the interpretation algorithm to explain the results.

To evaluate the performance of the proposed method, we use sensitivity, specificity, accuracy, F1, and AUC as our metrics. These metrics are defined as follows:

(9) $$\begin{array}{c} \text{sensitivity}=\frac{\text{TP}}{\text{TP}+\text{FN}},\\ \text{specificity}=\frac{\text{TN}}{\text{TN}+\text{FP}},\\ \text{accuracy}=\frac{\text{TP}+\text{TN}}{\text{TP}+\text{FN}+\text{TN}+\text{FP}},\\ \text{F}1=\frac{2∙\text{TP}}{2∙\text{TP}+\text{FP}+\text{FN}}, \end{array}$$

where TP is defined as the number of ASD subjects that are correctly classified, FP is the number of normal subjects that are misclassified as ASD subjects, TN is defined as the number of normal subjects that are correctly classified, and FN is defined as the number of ASD subjects that are misclassified as normal subjects. Specifically, sensitivity measures the proportion of ASD subjects that are correctly identified as such; specificity measures the proportion of normal subjects that are correctly identified as such. AUC is defined as the area under the Receiver Operating Characteristic (ROC) curve.

### 4.1. Comparison Models

Given the above FCNN model, we use the following models as benchmarks for comparison.

SVM: support-vector machine (SVM) model with linear kernel and rbf kernel. The SVM method has been widely used to classify fMRI data for brain disorders. The parameters are chosen by grid search.

RF: random forest (RF) is an ensemble learning method for classification. The parameters are chosen by grid search.

Autoencoder+MLP: the model was proposed by Sólon et al. [9]. Two stacked denoising autoencoders are pretrained; then, the encoder weights are applied to a multilayer perceptron (MLP), and the MLP is fine tuned to predict the probability of any given participants being ASD. We applied the encoder weights to the MLP with the configuration: 6670-1000-600-2.

ASD-DiagNet: this method is proposed by Eslami et al. [11]. An autoencoder is used to extract a lower dimensional feature representation. Then, the feature representation is fed into a single-layer perceptron (SLP) with sigmoid function for classification. The autoencoder and SLP classifier are trained simultaneously. The input layer and output layer have 6670 units fully connected to a bottleneck of 1667 units from the hidden layer. Data augmentation using EROS similarity measure is applied with 5 nearest neighbors of each sample.

FCNN: the proposed FCNN model as described above in Figure 2. The model contains two fully connected layers: the first layer has 64 units and the second layer has 32 units. The dropout ratio is set to 0.8. We used the Adam optimizer with a learning rate of 0.0005.

For autoencoder+MLP [9] and ASD-DiagNet [11], we used their online code to evaluate the models.

All functional connectivity features are flattened into one dimensional vector (see Figure 1), and the vectors are inputs in all model for training and classification. All the models were trained with 6670 functional connectivity features for each subject. We employed a 5-fold cross-validation setting to evaluate the performance of all the models. The experiments were carried out on all 871 subjects including both ASD patients and healthy controls.

### 4.2. Classification Results

**Figure 3:**
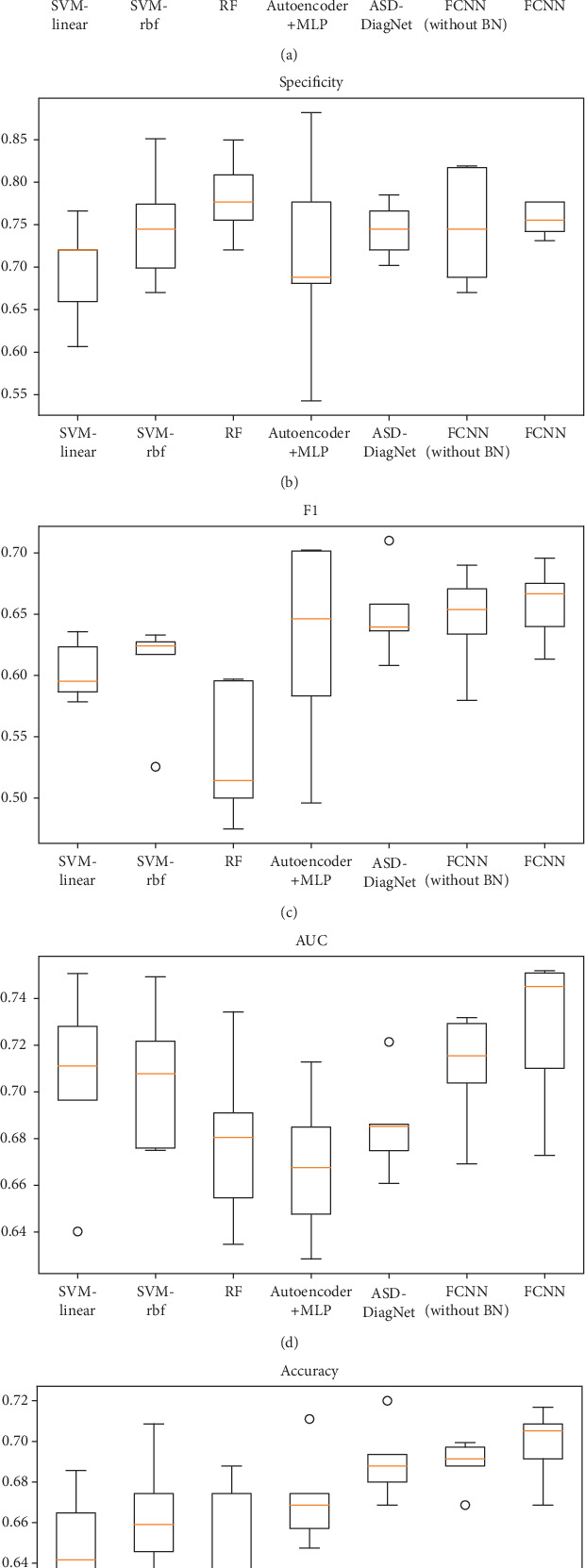
(a) Sensitivity, (b) specificity, (c) *F*1, (d) AUC, and (e) accuracy for classification task.

The classification results are shown in Table 1 and Figure 3. Box plots for sensitivity, specificity, F1, AUC, accuracy for classification task using 5-fold cross-validation are shown in Figure 3, where the middle line in each box represents the median value, and the circle represents the outlier.

The proposed FCNN model achieved the best performance on most evaluation metrics with accuracy of 69.81%, sensitivity of 63.05%, specificity of 75.63%, F1 of 65.82%, and AUC of 0.7262. The results showed that the deep learning models (FCNN, autoencoder+MLP, and ASD-DiagNet) have the better classification performance in general than the traditional methods (SVM and RF) on the resting-state fMRI dataset. As for the method autoencoder+MLP [9], we would like to mention that they reported 70% accuracy in their paper; the performance we reported is not as good as theirs, maybe because the brain atlas we used is different.

We also compared the FCNN model with or without the BN (Batch Normalization) layer in Table 1. The results showed that the BN layer improves the performance and stability of the model.

## 5. Interpretation Experiments and Analysis

### 5.1. Model Interpreting for an Instance

According to Section 3.3, for a trained FCNN model and any instance *x* with *n* features, *x* = {*x*_1_, *x*_2_, ⋯, *x*_*n*−1_, *x*_*n*_}, the fixed model can be formulated as a linear classifier with a fixed instance:

(10) $$\begin{array}{c} g\left(x\right)=\hat{W}x=w_{1}x_{1}+w_{2}x_{2}+\cdots +w_{n}x_{n}. \end{array}$$

Since the number of layers *L* is 4 for the FCNN model we used in this paper, so the weight vector $\hat{W}$ can be computed as

(11) $$\begin{array}{c} \hat{W}=\left(W^{\left(3\right)}\circ r^{\left(3\right)}\circ {\tilde{\gamma }}^{\left(3\right)}\right)\left(W^{\left(2\right)}\circ r^{\left(2\right)}\circ {\tilde{\gamma }}^{\left(2\right)}\right)W^{\left(1\right)}. \end{array}$$

**Figure 4:**
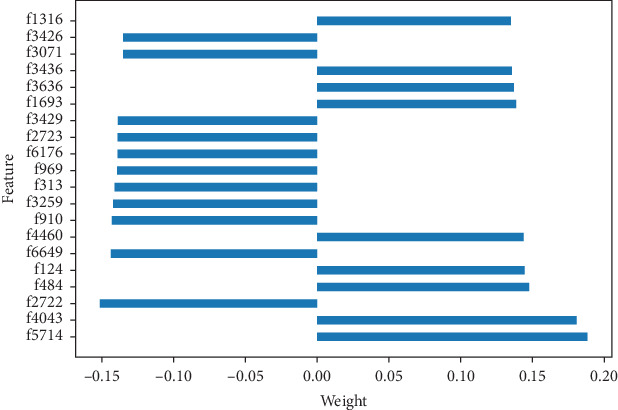
Weight visualization of some features of an instance.

The trained model could be interpreted with linear formula for any instance. Given an instance, we can get the weight of each feature from the trained model according to Equations (10) and (11). Some feature weights of an instance are visualized in Figure 4. The vertical axis represents the feature index, and the horizontal axis represents the weight value. It can help to understand the prediction result according to the feature index which can correspond to the brain region involved in the feature.

### 5.2. Model Interpreting for the ASD Group

Based on the trained FCNN model, we used Algorithm 1 as described in Section 3.3 to extract the decision features of the model. We set the top-important feature parameter *K* from 5 to 300, with an interval of 5, and the parameter *ε* as 95%, and then, we get a set of decision features with different *K*.

#### 5.2.1. Decision Feature Evaluation

**Figure 5:**
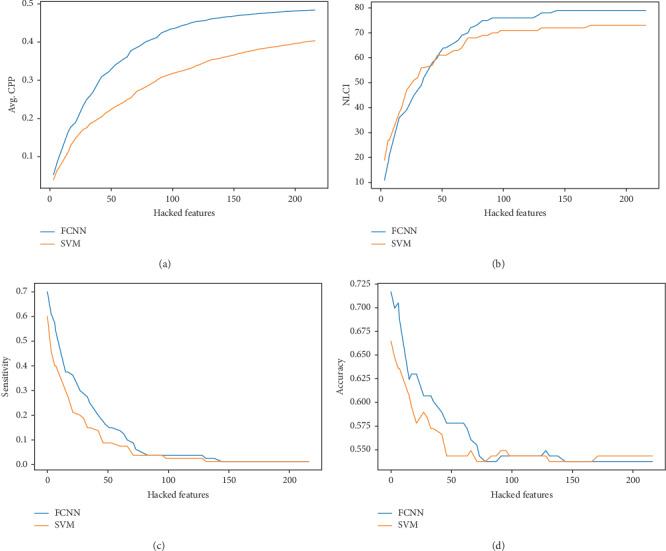
The performance of decision features on FCNN and SVM.

To evaluate the quality of the decision features, we analyzed the FCNN model by setting the values of the decision features in instance *x* to zero and observed the changes of prediction of FCNN. We used metrics including sensitivity, accuracy, and the change of prediction probability (CPP) which is the absolute change of probability of classifying *x* as a positive instance, the number of label-changed instance (NLCI) which is the number of instances whose predicted label changes after being hacked. For comparison, we also used the top *N* weighted features of linear-SVM to hack linear-SVM. The results are shown in Figure 5. It is shown that average CPP of FCNN is higher, and the NLCI of FCNN can be more than SVM with more decision features. And FCNN has considerable performance in sensitivity and accuracy.

For further comparison, we also applied the popular locally linear interpretation method (LIME) [27] to get the decision features in the trained FCNN model. Similar to Algorithm 1 in Section 3.3, we obtain the top *K* important features of each instance, and then, we obtain the decision feature set *F* by selecting the feature *f* whose occurrence number as a percentage of total instances is greater to the parameter *ε*. We set the same parameters (*K* from 5 to 300, with an interval of 5, and the parameter *ε* as 95%), and we did not obtain any decision feature. What is more, when we loosed the parameter *ε* to 20%, we also did not get any one feature. It means that the top 300 important features of the instance obtained by the LIME method are very different between instances in this model.

#### 5.2.2. Decision Feature Analysis

When *K* is taken as 20, 15 decision features were obtained; we selected these 15 decision features as a case for further analysis. There are 23 brain regions (ROIs) of the AAL atlas that involved these 15 rsFC connections. These 15 rsFCs and 23 ROIs are shown in Table 2.

We computed the mean value of each rsFC of the ASD group and the HC group, respectively, as well as the mean difference of two groups. An independent two-sample *t* test was run on the means of the rsFC elements of two groups. The analysis is shown in Table 2. Among these 15 rsFCs, 2 rsFCs are statistically significant (*p* < 0.05) between the ASD and HC groups, and the rest of rsFCs are not statistically significant. It demonstrates that FCNN could find underlying features though the feature values are not statistically different between groups.

**Figure 6:**
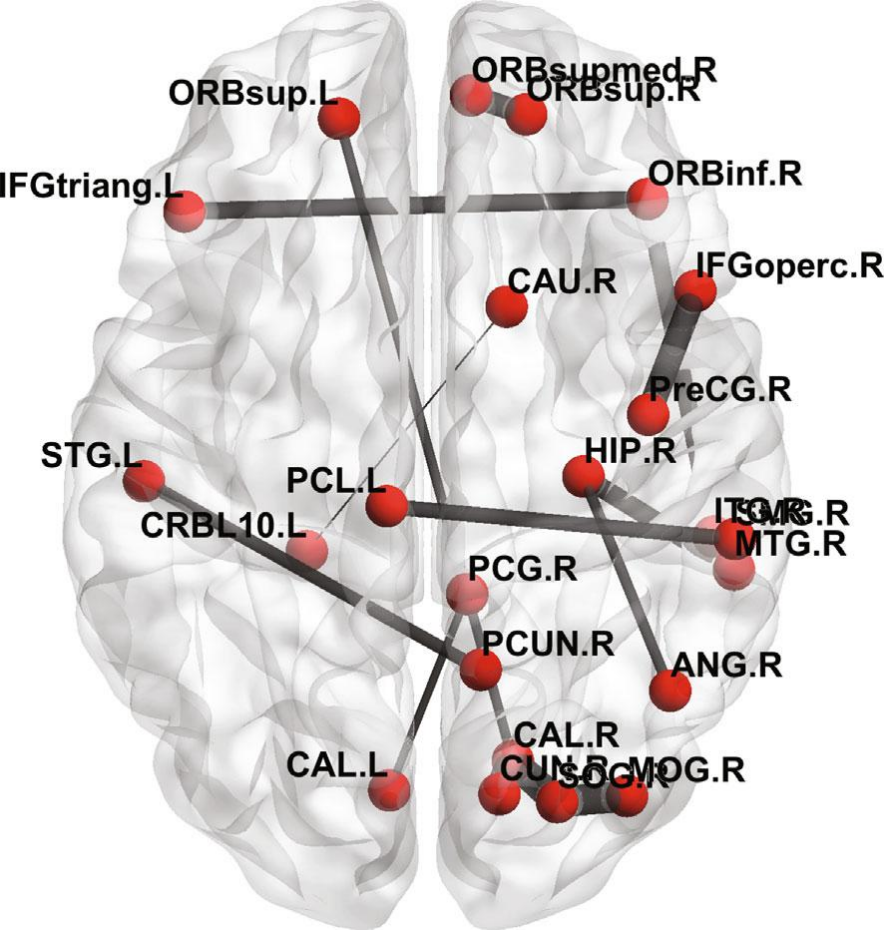
The visualization of 15 rsFCs from the ASD group.

These 15 rsFC connections of the AAL atlas are visualized in Figure 6, where the label information is from the AAL atlas. The thicker connection indicates two regions are strongly correlated and vice versa. The figure was drawn with BrainNet Viewer [28] software.

#### 5.2.3. Impact of Parameter *ε*

**Figure 7:**
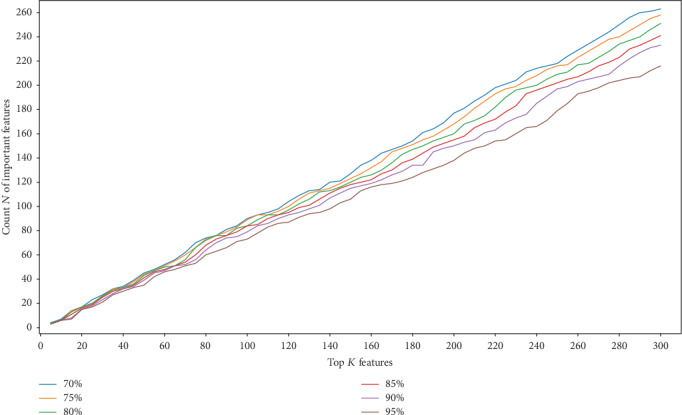
The number of decision features with different *K* and *ε*.

In order to evaluate the influence of parameter *ε* on the obtained decision features, we set the parameter *K* from 5 to 300, with an interval of 5, and the parameter *ε* from 70% to 95%, with an interval of 5%; then, *N* decision features were obtained accordingly. The result is shown in Figure 7. It is clear that the smaller the parameter *ε*, the more decision features will be obtained. While with a fixed *K*, the bigger the parameter *ε*, the fewer the decision features will be obtained.

## 6. Conclusion and Discussion

In this paper, we introduce an interpretable learning approach for resting-state functional connectivity analysis. We firstly propose an interpretable FCNN to classify ASD from HC, based on rsFC features. We experimentally compared the FCNN model against widely used classification models including SVM, RF, and two new classes of deep neural network models in a large dataset containing 871 subjects from ABIDE I database. The results show the proposed FCNN model achieves the highest classification accuracy 69.81%.

We further propose an interpreting method which could explain the trained model with a precise linear formula for each input instance and identify decision features of the model which contributed most to the classification of ASD versus HC participants.

Though being focused on ASD analysis in this presentation, the proposed approach could be generalized to benefit many other brain science and medicine applications that involve deep neural networks. Particularly, this study offers a promising deep learning-based approach to explore potential biomarkers for assisting brain neurological disorder diagnosis and research.

There are two limitations in the current work presented here. First, the dataset is limited to the 871 participants that contained ASD and HC. In order for this work to be more generalizable, it would be important to inspect and compare these initial findings with more fMRI data from more participants. Second, the proposed model is a compact fully connected neural network, given the number of layers and nodes in the model. Thus, it would be important to inspect the effectiveness of our interpreting approach for other types of neural network such as deeper and more complex architectures in the deep learning literature. Future work should focus on the accuracy and interpretation of our proposed approach for other large-scale fMRI data as well as other neuroimaging data based on brain disorders such as ASD.

## Figures and Tables

**Algorithm 1:**
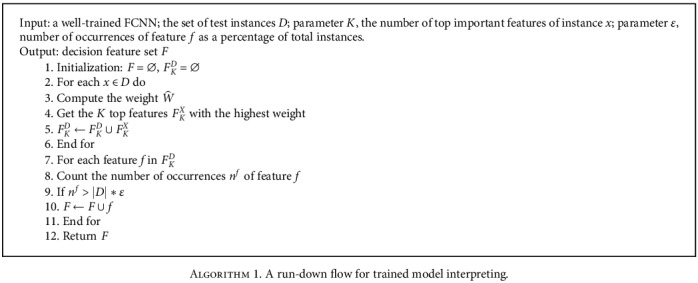
A run-down flow for trained model interpreting.

**Table 1:** Classification performance using 5-fold cross-validation (mean ± std).

	Accuracy	Sensitivity	Specificity	F1	AUC
SVM-linear	0.6441 ± 0.0281	0.5856 ± 0.0238	0.6946 ± 0.0556	0.6039 ± 0.0219	0.7053 ± 0.0372
SVM-rbf	0.6624 ± 0.0283	0.5631 ± 0.0623	0.7478 ± 0.0629	0.6055 ± 0.0403	0.7059 ± 0.0283
RF	0.6326 ± 0.0416	0.4590 ± 0.0428	0.7821 ± 0.0442	0.5364 ± 0.0506	0.6790 ± 0.0339
Autoencoder+MLP [9]	0.6717 ± 0.0217	0.6225 ± 0.1601	0.7140 ± 0.1124	0.6259 ± 0.0784	0.6682 ± 0.0293
ASD-DiagNet [11]	0.6900 ± 0.0172	0.6277 ± 0.0642	0.7436 ± 0.0299	0.6504 ± 0.0338	0.6857 ± 0.0201
FCNN (without BN)	0.6889 ± 0.0109	0.6204 ± 0.0844	0.7479 ± 0.0624	0.6456 ± 0.0378	0.7099 ± 0.0227
FCNN	0.6981 ± 0.0169	0.6305 ± 0.0474	0.7563 ± 0.0182	0.6582 ± 0.0287	0.7262 ± 0.0308

**Table 2:** Analysis of 15 most significant rsFCs.

Connection ID	ROI number	Regions	ASD mean conn	Control mean conn	Mean difference	*p* value
1	72	Caudate_R	0.0919	0.0728	0.0192	0.4390
	107	Cerebelum_10_L				
2	44	Calcarine_R	0.7370	0.7256	0.0114	0.4920
	46	Cuneus_R				
3	2	Precentral_R	0.5996	0.5474	0.0522	0.0325
	12	Frontal_Inf_Oper_R				
4	50	Occipital_Sup_R	0.7175	0.7136	0.0038	0.8445
	52	Occipital_Mid_R				
5	5	Frontal_Sup_Orb_L	0.2666	0.2309	0.0357	0.1985
	36	Cingulum_Post_R				
6	16	Frontal_Inf_Orb_R	0.4435	0.4311	0.0124	0.6172
	90	Temporal_Inf_R				
7	13	Frontal_Inf_Tri_L	0.4219	0.4192	0.0027	0.9201
	16	Frontal_Inf_Orb_R				
8	6	Frontal_Sup_Orb_R	0.5359	0.4989	0.0371	0.2355
	26	Frontal_Med_Orb_R				
9	44	Calcarine_R	0.6847	0.6632	0.0215	0.3427
	50	Occipital_Sup_R				
10	64	SupraMarginal_R	0.3853	0.3586	0.0267	0.3485
	69	Paracentral_Lobule_L				
11	38	Hippocampus_R	0.2871	0.2618	0.0253	0.3651
	66	Angular_R				
12	36	Cingulum_Post_R	0.2296	0.2110	0.0185	0.5694
	43	Calcarine_L				
13	36	Cingulum_Post_R	0.2640	0.2474	0.0167	0.6089
	44	Calcarine_R				
14	38	Hippocampus_R	0.4710	0.4123	0.0586	0.0377
	86	Temporal_Mid_R				
15	68	Precuneus_R	0.3425	0.3111	0.0314	0.2958
	81	Temporal_Sup_L				

## Data Availability

The data used to support the findings of this study are available from the corresponding author upon request. The ABIDE I dataset analyzed during this study is available in the Preprocessed Connectomes Project website (http://preprocessed-connectomes-project.org/abide/download.html).
